# Understanding mechanisms of reduced cefiderocol susceptibility in *pvdS* and *fecI* mutants of *Pseudomonas aeruginosa*

**DOI:** 10.1093/jac/dkag004

**Published:** 2026-01-30

**Authors:** Yoshinori Yamano, Miki Takemura, Christopher Longshaw, Boudewijn L M DeJonge, Naoki Ishibashi, Ryuta Ishii, Dai Miyagawa, Rio Nakamura, Miho Kuroiwa, Yoshino Ishioka, Takumi Adachi, Masatomo Rokushima, Hideki Maki, Takafumi Sato

**Affiliations:** Drug Discovery Research Division, Shionogi & Co., Ltd., Toyonaka, Osaka 561-0825, Japan; Drug Discovery Research Division, Shionogi & Co., Ltd., Toyonaka, Osaka 561-0825, Japan; Scientific Affairs, Shionogi B.V., London, UK; US Medical Affairs, Shionogi Inc., Florham Park, NJ, USA; Drug Discovery Research Division, Shionogi & Co., Ltd., Toyonaka, Osaka 561-0825, Japan; Drug Discovery Research Division, Shionogi & Co., Ltd., Toyonaka, Osaka 561-0825, Japan; Drug Discovery Research Division, Shionogi & Co., Ltd., Toyonaka, Osaka 561-0825, Japan; Drug Discovery Research Division, Shionogi & Co., Ltd., Toyonaka, Osaka 561-0825, Japan; Drug Discovery Research Division, Shionogi & Co., Ltd., Toyonaka, Osaka 561-0825, Japan; Drug Discovery Research Division, Shionogi & Co., Ltd., Toyonaka, Osaka 561-0825, Japan; Drug Discovery Research Division, Shionogi & Co., Ltd., Toyonaka, Osaka 561-0825, Japan; Drug Discovery Research Division, Shionogi & Co., Ltd., Toyonaka, Osaka 561-0825, Japan; Drug Discovery Research Division, Shionogi & Co., Ltd., Toyonaka, Osaka 561-0825, Japan; Drug Discovery Research Division, Shionogi & Co., Ltd., Toyonaka, Osaka 561-0825, Japan

## Abstract

**Objectives:**

Understand how mutations upstream of *pvdS* and *fecI* in *Pseudomonas aeruginosa* PAO1 lead to reduced susceptibility to cefiderocol, a siderophore cephalosporin.

**Materials and methods:**

Whole-genome sequencing, gene complementation and inactivation, mRNA expression, siderophore quantification and supplementation were carried out with mutants that showed reduced susceptibility to cefiderocol.

**Results:**

Whole-genome sequencing, combined with gene complementation studies, with isolated mutants that showed 4-fold reduced susceptibility to cefiderocol identified mutations within the Fur-box of promoter regions upstream of *pvdS* and *fecI*. The *pvdS* promoter mutation led to increased *pvdS* transcription, resulting in increased pyoverdine production, as well as reduced transcription of other iron transport-related genes, including *piuA* an iron transporter responsible for cefiderocol uptake. The down-regulation of *piuA,* rather than competition for iron between pyoverdine and cefiderocol, was responsible for the decreased cefiderocol susceptibility. The *fecI* mutant demonstrated increased transcription of *fecI* and *fecA*, and the expression level of *piuA* in the *fecI* mutant was not related to reduced cefiderocol susceptibility, indicating a different mechanism for the reduced cefiderocol susceptibility in this mutant. Sequence analyses of over 35 000 clinical *P. aeruginosa* isolates did not identify isolates with the *pvdS* mutation, and only one isolate with the *fecI* mutation.

**Conclusions:**

These data highlight different mechanisms by which *P. aeruginosa* attempts to evade the action of cefiderocol by exploiting the complexities of iron haemostasis in bacteria. The absence of such mutants in the clinic suggests that such mutations may be detrimental for *P. aeruginosa* to survive in the clinical environment.

## Introduction

Cefiderocol is a siderophore cephalosporin that uses iron uptake systems to penetrate through the outer membrane of Gram-negative bacteria and shows good activity against those pathogens, including resistant isolates.^1,2^ Activity of cefiderocol against *P. aeruginosa* was confirmed in large surveillance studies, where 98% of the >10 000 isolates tested as susceptible according to Clinical and Laboratory Standards Institute (CLSI) or European Committee on Antimicrobial Susceptibility Testing (EUCAST) breakpoints.^3,4^

There have been several reports on mechanisms that can reduce susceptibility to cefiderocol in *P. aeruginosa*. *in vitro* studies showed that a deficiency of the iron transporters PiuA or PiuD, which are used for the outer membrane penetration of cefiderocol, resulted in reduced susceptibility for cefiderocol.^5,6^ Reduced susceptibility has also been reported as a result of the production of several β-lactamases such as SHV-12, KPC with Ω-loop mutations, NDMs and OXA-15 or specific mutations in *ampC* (e.g. E247K, L293P) which all lead to enhanced cefiderocol hydrolysis.^7^ Clinical studies also implicated mutations in *ampC* or *ampD* combined with mutations in the iron-transporter genes such as *piuA* and *piuD*.^8–10^ Recently, Galdino *et al.* showed that pyoverdine production correlated with reduced cefiderocol susceptibility, which was attributed to stripping iron from cefiderocol, thereby limiting its uptake.^11^

In this study, mutations in the upstream regions of *pvdS* and *fecI* were identified that lead to reduced susceptibility to cefiderocol in *P. aeruginosa* PAO1. PvdS and FecI are extracytoplasmic function (ECF) sigma factors that are controlled by Fur, which acts as a repressor when bound to its co-repressor Fe^2*+*^, regulating the expression of proteins involved in iron metabolism.^12,13^ They regulate expression of iron uptake systems, which in general consist of siderophores that can strip iron bound to host proteins, and iron uptake systems that allow for the uptake of the iron-bound siderophore to supply the bacterial cell with iron that is essential for growth.^14^ Besides the regulation of iron transport-related genes, PvdS also regulates the expression of several virulence factors.^12,13^ Studies were undertaken to understand how the mutations in the promoter regions of *pvdS* and *fecI* can lead to reduced cefiderocol susceptibility in *P. aeruginosa* PAO1.

## Materials and methods

### Bacterial strains, antibiotics, pyoverdines and plasmids

*P. aeruginosa* PAO1 was kindly provided by Tokai University. *P. aeruginosa* strains PW8599 (PAO1 with transposon insertion in *piuA*) and PW5085 (PAO1 with transposon insertion in *pvdS*) were kindly provided by the University of Washington.^15^ Strain SR-L00016 (PW8599 with *pirA* deletion) was constructed previously.^5^

The siderophore β-lactams cefiderocol (a cephalosporin with a catechol residue as the iron-chelating group), SMC-3176, MB-1, and BAL30072 (all monocyclic β-lactams with hydroxypyridone residues as iron-chelating group) were synthesized at the research laboratories of Shionogi & Co., Ltd (Japan).^16–18^ Ceftazidime and piperacillin-tazobactam were obtained from Chem-Impex International, Inc. (USA), ciprofloxacin and amikacin from LKT Laboratories, Inc. (USA) and meropenem from Tokyo Chemical Industry Co., Ltd (Japan). All acquired antibiotics were commercial grade, and all antibiotics were solubilized and diluted in water to obtain the appropriate concentrations. Three types pyoverdines, pyoverdine ATCC 15692 (pyoverdine I), pyoverdine ATCC 27853 (pyoverdine II) and pyoverdine Pa 6 (pyoverdine III) were obtained from EMC microcollections (Germany).^19^ Pyoverdines were solubilized in a small amount of dimethyl sulfoxide, followed by dilutions in water to obtain the appropriate concentrations.

Recombinant plasmids for the expression of *pvdS*, *fecI* or *fecA* were constructed by using pMMB67HE/Gm, a derivative of pMMB67HE (American Type Culture Collection, USA) in which the ampicillin resistance gene was replaced by the gentamicin resistance gene, because *P. aeruginosa* PAO1 is intrinsically resistant to ampicillin. The gentamicin resistance cassette on plasmid pFastBac Dual (Thermo Fisher, USA) was amplified with the primers Gm-In-F and Gm-In-R (Table S1, available as Supplementary data at *JAC* Online), and was used to substitute the *bla* region of plasmid pMMB67HE by In-Fusion^®^ HD Cloning Kit w/Cloning Enhancer (Takara Bio, Japan), producing plasmid pMMB67HE/Gm. *pvdS*, *fecI* or *fecA* were amplified with primers from *P. aeruginosa* PAO1 (Table S1), and each were inserted into pMMB67HE/Gm digested with *Bam*HI and *Hin*dIII, resulting in recombinant plasmids ppvdS, pfecI or pfecA, respectively. The recombinant plasmid ppiuA for the expression of *piuA* was constructed previously using the same method.^5^

### Minimum inhibitory concentration

Minimum inhibitory concentration (MIC) of cefiderocol and other antibiotics was determined using broth microdilution according to CLSI procedures.^20^ For the determination of MIC of cefiderocol and the other siderophore β-lactams (MB-1, SMC-3176 and BAL30072), iron-depleted cation-adjusted Mueller–Hinton broth (ID-CAMHB) was used, which was prepared by removing iron from BBL Mueller–Hinton broth (Becton Dickinson and Company, USA) with the cation-binding Chelex resin (Bio-Rad, USA).^20,21^ MICs were also determined in the presence of pyoverdine at 1 or 10 μM, corresponding to the concentration of pyoverdine I produced by *P. aeruginosa* PAO1 and mutant SR100213, respectively (see below). Cefiderocol MICs against the strains harbouring expression plasmids were determined in ID-CAMHB supplemented with 0.1 mM IPTG to induce the gene expression from the recombinant plasmids.

### Selection of spontaneous mutants with decreased susceptibility to cefiderocol

An overnight culture of *P. aeruginosa* PAO1 was diluted into fresh medium to yield 1.4 × 10^9^ cfu/mL. Aliquots of 0.1 mL were plated onto Mueller–Hinton agar (MHA) (Becton Dickinson and Company, USA) containing 4× or 10 × MIC of cefiderocol and incubated at 37°C for 48 h. Colonies growing on MHA containing cefiderocol were serially transferred on MHA without cefiderocol for 5 days after which the MIC values were determined as described before. Mutants with elevated MIC values were frozen in Mueller–Hinton broth containing 20% glycerol for storage and used in subsequent studies.

### Whole-genome sequencing

Genomic DNA was extracted from overnight cultures using the Illustra bacteria genomic Prep Mini Spin Kit (GE Healthcare, USA), and unique index-tagged libraries were prepared with the Nextera XT DNA Sample Prep Kit (Illumina, Inc., USA) according to the manufacturer’s instructions. Whole-genome sequencing (WGS) was performed on the MiSeq system (Illumina, Inc., USA) with 300-base paired-end reads. The FASTQ reads were trimmed and assembled into contigs for each test strain using CLC Genomics workbench v.9.5.3 (CLC Gx) (Qiagen, USA). To detect genomic mutations in individual isolates, trimmed reads were mapped to the reference genomes and variant calling was performed using CLC Gx. Called variants were compared between the parent and mutants using Pipeline Pilot v,9.5 (PP) (Dassault Systèmes BIOVIA, USA). The detected mutations were confirmed by Sanger sequencing with BigDye Terminator v,3.1 (Thermo Fisher Scientific, USA).

### Prevalence of *fecI* and *pvdS* mutations in clinical isolates

Genome sequence data of *P. aeruginosa* were retrieved from the NCBI Pathogen Detection database by specifying ‘*Pseudomonas aeruginosa*’ as the taxgroup_name, and clinical isolates were identified by selecting ‘clinical’ in the ‘Isolation type’ column. FASTA-formatted genome sequences of clinical *Pseudomonas aeruginosa* isolates were downloaded. BLASTN searches were performed against these genomes using the *pvdS* and *fecI* loci from *P. aeruginosa* PAO1 as queries, with promoter regions included (125 bp upstream of *pvdS* start codon and 83 bp upstream of *fecI* start codon). Genomic regions identified by the BLASTN search were extracted from each genome, and multiple sequence alignment was performed using the MAFFT algorithm to identify the nucleotide polymorphisms in the upstream regions of *pvdS* and *fecI* (−83 C > A, for *pvdS*; −44_45 insA for *fecI*).

### Determination of mRNA expression

Expression of *pvdS* and *fecI* was measured to determine the effect of the mutation in the corresponding promoter region on the expression level of each gene. The expression of *piuA* was also determined, as gene-knockout experiments have shown PiuA to be a main iron uptake transporter in *P. aeruginosa* PAO1 involved in cefiderocol uptake.^5^ The expression of *fecA* was determined as it is regulated by FecI.

*P. aeruginosa* PAO1, grown overnight in ID-CAMHB, was diluted in ID-CAMHB and grown for 8 hours to reach mid-exponential growth phase (OD_600_ = 0.2). Cells were concentrated and RNA was extracted with RNeasy mini kit (Qiagen, USA) and converted to single-stranded cDNA with High-Capacity cDNA Reverse Transcription Kit (Thermo Fisher Scientific, USA) according to the manufacturer’s instruction. Real-time RT–qPCR was conducted with Power SYBR Green PCR Master Mix (Thermo Fisher Scientific, USA) and Applied Biosystems^TM^ 7500 Fast Real-Time PCR System (Thermo Fisher Scientific, USA) according to the manufacturer’s instruction. *uvrD* was chosen as a reference gene for normalization of real-time RT–qPCR data^22^ (Table S1). The quantification of target genes was performed by extracting RNA three times from one culture and calculating the average value ± SD of these three determinations. Transcription analyses of each gene were performed by ddCT method, and the quantities of target gene transcripts in the strain PAO1 were each assigned to a value of 1.0.

### Semi-quantitative measurement of pyoverdine I

Production of pyoverdine I, the only pyoverdine produced by *P. aeruginosa* PAO1 and its mutants,^15^ was determined in the supernatant of overnight cultures of *P. aeruginosa* grown in ID-CAMHB by mass spectrometry. Pyoverdine II was used as an internal standard, to standardize matrix effect and sample loss at the preparation step.

Supernatants were acidified to pH 3 to 4 with 90% formic acid and diluted, followed by the addition of 50 μM pyoverdine II. Solid-phase extraction was performed using Oasis MCX 96-well µElution Plate (Waters, USA). The samples were applied to the plate and washed with water and methanol, followed by elution with 20% acetonitrile containing 8% NH_3_. The eluate was acidified with 90% formic acid, and loaded onto µFocus MALDI sample plates (Hudson Surface Technology, USA) pre-coated with α-cyano-4-hydroxycinnamic acid matrix. An Ultraflex MALDI TOF mass spectrometer (Bruker Daltonics, USA) was used in Reflector positive ion mode to determine the peak areas of pyoverdine I (*m/z* 1333.7) and pyoverdine II (*m/z* 1092.5). The relative amount of pyoverdine of PAO1 and its mutants was determined by the peak area ratio of pyoverdine I (*m/z* 1333.7) to internal standard (*m/z* 1092.5), which was divided by OD_600_ of each culture and normalized to PAO1 (Table S2).

### Assessment of lethality using a neutropenic murine systemic infection model

JCl:ICR male mice with weight of 17 to 20 g (CLEA Japan Inc., Japan) were rendered neutropenic by intraperitoneal administration of cyclophosphamide (Shionogi, Japan) with 150 and 100 mg/kg 4 days and 1 day before infection, respectively. Mice were infected intraperitoneally with bacterial suspensions in 0.5 mL of mucin (*n* = 5 for each dose). The 50% lethality doses (LD_50_s) were calculated by the logit method using the number of surviving mice 7 days after infection with the Windows SAS program (SAS^®^ v.9.4). The experiment was conducted in triplicate independently, and geometric averages and coefficient of variation (CV) of these three experiments were calculated.

## Results and discussion

### Isolation and characterization of mutants of *P. aeruginosa* PAO1 with decreased susceptibility to cefiderocol

Spontaneous mutants from *P. aeruginosa* PAO1 with decreased susceptibility to cefiderocol were selected by spreading cells on agar plates containing 4× or 10 × MIC of cefiderocol. After incubation at 37˚C for 48 hours, no colonies appeared on the plates containing 10 × MIC of cefiderocol, however, five colonies appeared on one plate containing 4 × MIC of cefiderocol. After serial passage on drug-free agar, four showed a stable resistance phenotype (designated as SR100213, SR100215, SR100216 and SR100217) with a 4-fold increase in cefiderocol MIC from 0.5 to 2 mg/L. WGS showed that three mutants (SR100213, SR100215 and SR100217) all had the same nucleotide substitution of C to A located at −83 nucleotide upstream from the *pvdS* coding region, corresponding to the putative ferric uptake regulator binding site (Fur-box) within the promoter region of *pvdS*, which encodes an ECF sigma-70 factor that controls expression of genes responsible for the biosynthesis of the siderophore pyoverdine.^23–27^ The remaining mutant SR100216 had an adenine insertion at −44 nucleotides upstream from the *fecI* coding region, corresponding to the putative Fur-box within the promoter region of *fecI*, which encodes an ECF sigma factor that regulates expression of the ferric citrate transporter FecA.^28^ Given the mutations, it was not unexpected that there was no significant change in susceptibility to other antibiotics (ceftazidime, meropenem, piperacillin/tazobactam, ciprofloxacin and amikacin) for these mutants, but that there were MIC increases for other siderophore β-lactam antibiotics such as MB-1, SMC-3176 and BAL30072 (Table 1).

**Table 1.** MIC values of *P. aeruginosa* PAO1 mutants with mutations in iron-transporter genes

Strains	Supplement (10 μM)	MIC (mg/L) (fold change compared with PAO1)									Mutation
		cefiderocol	MB-1	SMC-3176	BAL 30 072	ceftazidime	meropenem	piperacillin-tazobactam	ciprofloxacin	amikacin	
PAO1	—	0.5	0.125	0.125	0.5	1	0.5	2	0.125	4	Wild type
SR100213	—	2 (4)	16 (128)	4 (32)	>32 (>64)	1 (1)	0.5 (1)	4 (2)	0.125 (1)	4 (1)	Upstream region of *pvdS* (c.-83 C>A)
SR100216	—	2 (4)	8 (64)	8 (64)	8 (16)	1 (1)	1 (2)	4 (2)	0.125 (1)	4 (1)	Upstream region of *fecI* (c.-44_45 insA)
PW5805	—	0.125 (1/4)	NT	NT	NT	1 (1)	NT	NT	NT	NT	Tn insertion in *pvdS*
PAO1	Pyoverdine I-	4 (8)	32 (256)	16 (128)	>32 (>64)	1 (1)	NT	NT	NT	NT	Wild type
PW8599	—	2 (4)	4 (32)	2 (16)	2 (4)	2 (2)	NT	NT	NT	NT	Tn insertion in *piuA*
SRL-00016	—	2 (4)	16 (128)	16 (128)	>32 (>64)	2 (2)	NT	NT	NT	NT	Tn insertion in *piuA* and deletion of *pirA*

NT, not tested; c, coding DNA.

The prevalence of these mutations in clinical isolates was determined by analysing the genomes of *P. aeruginosa* available in the NCBI Pathogen Detection database. Of the 50 181 *P. aeruginosa* identified, 36 326 were annotated as clinical isolates and had genome sequence data available. Of these, 35 913 and 36 308 samples contained sequence data for the promoter region of *pvdS* and *fecI*, respectively. Nucleotide polymorphism analysis showed that there were no clinical strains with the cytosine to adenine mutation in the upstream region of *pvdS*, and there was only one sample with the adenine insertion in the upstream region of *fecI*, indicating that the mutations obtained in laboratory experiments are rarely or not encountered in clinical isolates.

#### Effect of mutations in the upstream region of *pvdS* on the expression of pyoverdine and iron uptake-related genes

**Figure 1.**
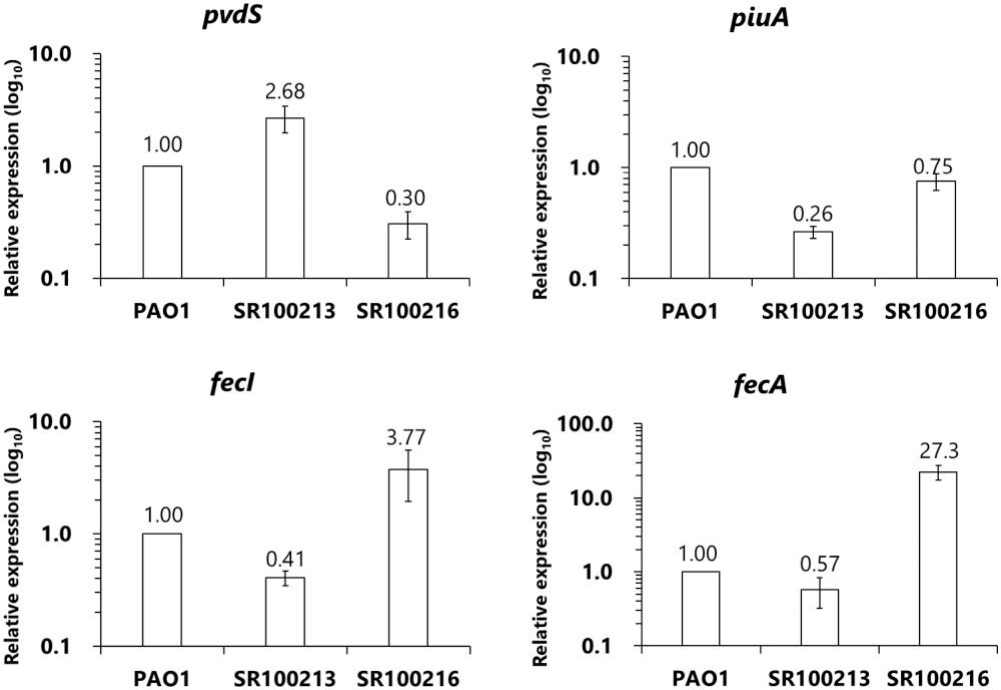
mRNA expression levels of iron uptake-related genes in mutants with reduced susceptibility to cefiderocol. mRNA expressions for *pvdS*, *piuA*, *fecI* and *fecA* genes of *P. aeruginosa* PAO1 (parent), SR100213 (a *pvdS* mutant) and SR100216 (a *fecI* mutant) were determined by real-time RT–PCR.

**Figure 2.**
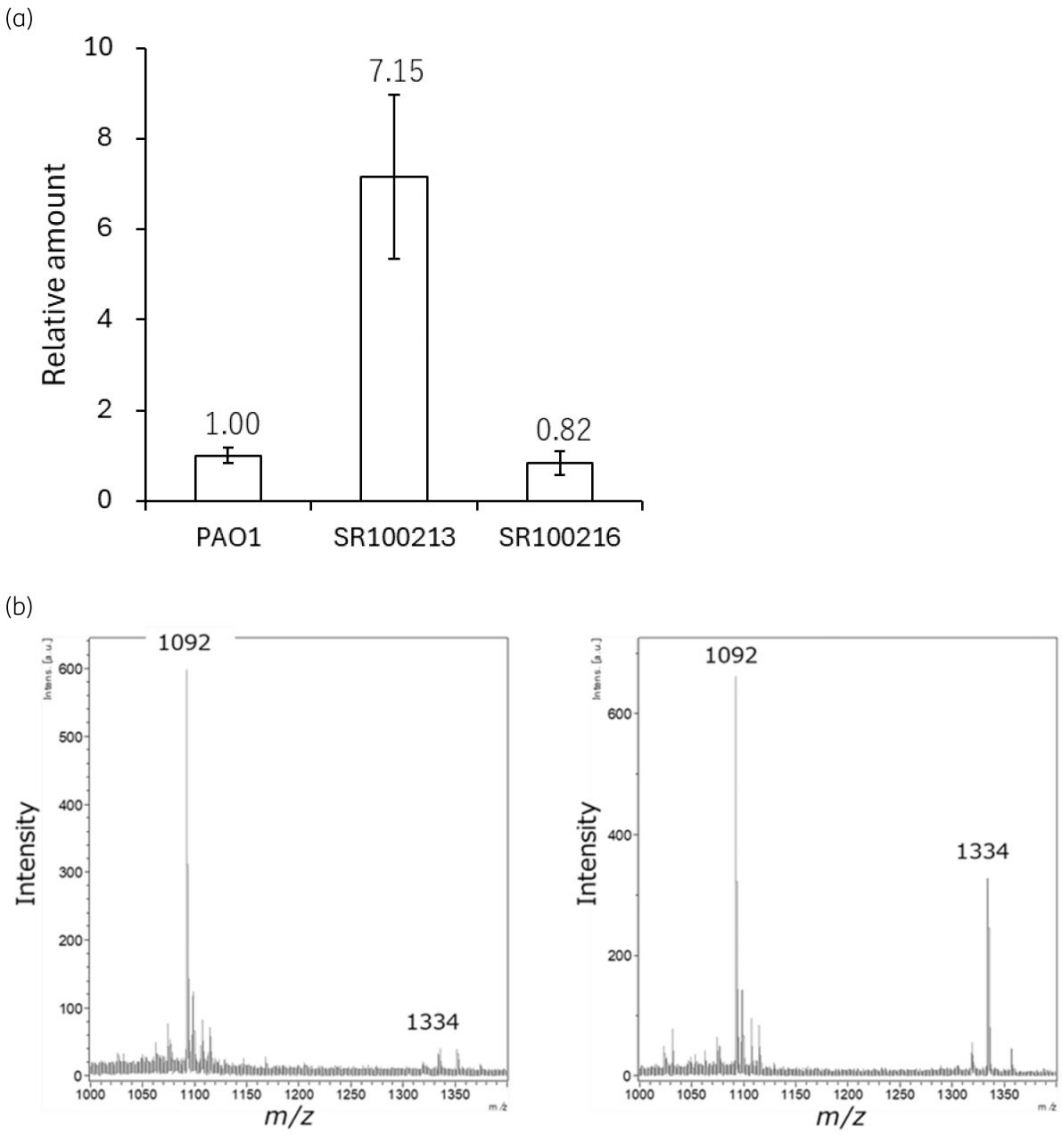
Pyoverdine I production in the mutants with reduced susceptibility to cefiderocol. (a) Production of the endogenous siderophore, pyoverdine I in the supernatant of overnight cultures of *P. aeruginosa* PAO1 (parent), SR100213 (a *pvdS* mutant) and SR100216 (a *fecI* mutant) were determined by using mass spectrometry. Pyoverdine I and pyoverdine II were used as a standard and an internal standard for the determination of the pyoverdine I production from PAO1 and its mutants. (b) MALDI-TOF/TOF/MS Spectra profile of the supernatant of strain PAO1 grown in iron-sufficient medium (left) and iron-depleted medium (right). The peak corresponding to molecular weight of 1334 and 1092 corresponded to pyoverdine I produced by strain PAO1 and pyoverdine II (50 μM) as an internal standard, respectively.

The expression of *pvdS* mRNA of mutant SR100213 was 2.68-fold higher than that of the parental strain PAO1 (Figure 1), suggesting that the mutation identified in the putative Fur-box of the *pvdS* gene affects Fur binding to the Fur-box, leading to increased expression of the *pvdS* gene, which has been reported to cause up-regulation of pyoverdine biosynthesis genes.^23^ SR100213 showed indeed an increased production of pyoverdine I compared with the parental strain PAO1, which was 7.15-fold increased and amounted to about 10 μM of pyoverdine I produced (Figure 2). Interestingly, a decreased mRNA expression of iron-transporter genes *piuA* and *fecA* as well as *fecI*, which regulates *fecA* expression, was also observed in SR100213 (Figure 1). As PiuA is an iron transporter responsible for the outer membrane transport of siderophore-conjugated β-lactams, including cefiderocol,^16,29,30^ the down-regulation of this particular iron transporter might result in reduced susceptibility to cefiderocol, rather than the overproduction of pyoverdine (see below).

The contribution of *pvdS* overexpression on reduced cefiderocol susceptibility was confirmed with a recombinant *P. aeruginosa* PAO1 that contained a plasmid that overexpresses *pvdS* (ppvdS) and showed reduced susceptibility to cefiderocol and other siderophore-conjugated β-lactams (Table 2). Furthermore, increased susceptibility to cefiderocol was observed for a transposon mutant of *pvdS* in strain PAO1 (strain PW5805), supporting the contribution of *pvdS* expression to cefiderocol susceptibility (Table 1). Likewise, the addition of pyoverdine I, the pyoverdine subtype produced by *P. aeruginosa* PAO1, at 10 μM (similar to the pyoverdine production level in SR100213) into the medium of *P. aeruginosa* PAO1 led to an 8-fold MIC increase of cefiderocol and >64-fold MIC increase of the other siderophore β-lactams (Table 1). Such MIC increases were also observed by the addition of 1 μM pyoverdine I (Table 3).

**Table 2.** Effect of overexpression of *pvdS*, *fecI* or *fecA* on the susceptibility of siderophore β-lactams

Strain/plasmid	MIC (mg/L) (fold change compared with vector for each strain)				
	cefiderocol	MB-1	SMC-3176	BAL30072	ceftazidime
PAO1/pMMB67HE/Gm	0.5	0.125	0.125	0.5	2
PAO1/ppvdS	4 (8)	16 (128)	8 (64)	>32 (>64)	2 (1)
PAO1/pfecI	4 (8)	8 (64)	4 (32)	8 (16)	2 (1)
PAO1/pfecA	4 (8)	4 (32)	4 (32)	16 (32)	2 (1)
SR100213/pMMB67HE/Gm	2	NT	NT	NT	NT
SR100213/ppiuA	0.5 (1/4)	NT	NT	NT	NT
SR100216/pMMB67HE/Gm	2	NT	NT	NT	NT
SR100216/ppiuA	2 (1)	NT	NT	NT	NT
SR100216/ppvdS	2 (1)	NT	NT	NT	NT

ppvdS, pfecI, pfecA and ppiuA are the recombinant plasmids that caused overexpression of *pvdS*, *fecI*, *fecA* and *piuA*, respectively, on the plasmid pMM67HE/Gm.

NT, not tested.

**Table 3.** Effect of exogenous pyoverdine I on the activity of siderophore β-lactams

Strain	MIC (mg/L) in the presence of pyoverdine I (0, 1 and 10 μM) (relative ratio of MIC by the addition of pyoverdine)													
	cefiderocol			MB-1			SMC-3176			BAL30072			ceftazidime	
	0	1	10	0	1	10	0	1	10	0	1	10	0	10
PAO1	0.5	2 (4)	4 (8)	0.125	4 (32)	32 (256)	0.125	2 (16)	16 (128)	0.5	16 (32)	>32 (>64)	1	1 (1)
PAO1Δ*piuA*	2	4 (2)	4 (2)	4	16 (4)	32 (8)	2	16 (8)	16 (8)	2	>32 (>16)	>32 (>16)	2	2 (1)
SR100213	2	4 (2)	4 (2)	16	16 (1)	32 (2)	4	16 (4)	32 (8)	>32	>32 (−)	>32 (−)	1	1 (1)
SR100216	2	4 (2)	4 (2)	8	8 (1)	32 (4)	4	8 (2)	16 (4)	>32	32 (−)	32 (−)	2	2 (1)

#### Mechanism of overproduction of pyoverdine on susceptibility to cefiderocol

Two possible mechanisms for the reduced susceptibility to cefiderocol due to overproduction of pyoverdine could be envisioned. First, pyoverdine overproduction could directly interfere with the formation of the iron-chelating complex with cefiderocol by competing for iron. Alternatively, pyoverdine overproduction could lead to an influx of the iron-pyoverdine complex, increasing the intracellular iron pool resulting in a down-regulation of expression of other iron transporters that facilitate the active transport of siderophore β-lactams.

To determine which of the previously mentioned mechanisms is in play, 10 μM of pyoverdine I, II and III was added to pyoverdine I or II producers to study the effect on cefiderocol susceptibility. Each strain of *P. aeruginosa* produces only one type of pyoverdine and its corresponding cognate iron-pyoverdine transporter.^19^ Addition of exogenous pyoverdine I to pyoverdine I producers caused an increase in the MIC of cefiderocol, but not for pyoverdine II producers (Table 4). Similarly, addition of exogenous pyoverdine II to pyoverdine II producers caused an increase in MIC of cefiderocol, but not for pyoverdine I producers. Addition of pyoverdine III did not cause an increase in MIC of cefiderocol for either pyoverdine I or II producers. These results show that overproduction of pyoverdine itself does not lead to direct antagonism with cefiderocol via competition for free iron, suggesting that the indirect down-regulation of the siderophore uptake transporters required for cefiderocol uptake is the likely reason for the reduced cefiderocol susceptibility. Galdino *et al.* reported that the addition of enterobactin to medium caused reduced cefiderocol susceptibility, which was explained by the competition between enterobactin and cefiderocol to chelate iron.^11^ However, because enterobactin can be used by *P. aeruginosa* to obtain iron,^31^ it is likely that addition of enterobactin to the medium results in an increased intracellular iron concentration, and as a consequence down-regulating the expression of *piuA*, thereby reducing uptake of the iron–cefiderocol complex, similar to what we observed for pyoverdine.

**Table 4.** Effect of exogenous pyoverdine on the activity of cefiderocol

Strain	Pyoverdine type^a^	MIC (mg/L) of cefiderocol (fold change compared with no addition) in the presence of 10 μM of			
		None	Pyoverdine I	Pyoverdine II	Pyoverdine III
PAO1	Pyoverdine I	0.125	1 (8)	0.25 (2)	0.125 (1)
SR24		0.125	2 (16)	0.25 (2)	0.25 (2)
DM3355		0.5	4 (8)	1 (2)	1 (2)
ATCC 27 853	Pyoverdine II	0.25	0.125 (0.5)	2 (8)	0.25 (1)
SR27000		1	1 (1)	8 (8)	1 (1)
SR08641		1	0.5 (0.5)	16 (16)	1 (1)

^a^Pyoverdine type produced by each strain.

To confirm that lower expression of *piuA* affected the susceptibility to cefiderocol in mutant SR100213, the mutant was complemented with plasmid ppiuA to overexpress *piuA*. Incorporation of the ppiuA plasmid resulted in an MIC decrease of SR100213 from 2 to 0.5 mg/L, strongly supporting that reduced PiuA production in the *pvdS* mutant SR100213 led to reduced cefiderocol susceptibility (Table 2). The fact that the MIC values for cefiderocol and other siderophore β-lactams of the *pvdS* mutant are similar to those of PAO1 isogenic mutant strain SRL-00016 with knock-out mutations in both *piuA* and *pirA*, is also supportive that the mutation in the upstream region of *pvdS* caused down-regulation of all iron transporters (Table 1).

PvdS is also involved in the regulation of various virulence-related genes such as exotoxin A and the PrpL protease.^13^ To determine whether the mutation in the promoter region of *pvdS* also affected virulence, the lethality dose of *P. aeruginosa* PAO1 and SR100213 was determined in a systemic infection mouse model. The LD_50_s for *P. aeruginosa* PAO1 and SR100213 were 1.6 × 10^4^, 1.2 × 10^5^, 4.1 × 10^4^ and 1.0 × 10^4^, 1.1 × 10^5^, 1.8 × 10^4^ cfu/mouse, respectively, with an average of 4.3 × 10^4^ cfu/mouse (CV, 133%) and 2.7 × 10^4^ cfu/mouse (CV, 194%) for strain PAO1 and SR100213, respectively. While the *pvdS* mutation did not show a significant difference in virulence in this *in vivo* model, production of toxins could still have been affected but this was not evaluated.

#### Effect of mutations in the upstream region of *fecI* on the expression of iron uptake-related genes

The *fecI* mutant SR100216 showed down-regulation of *pvdS* and *piuA* by 70% and 25%, respectively, and pyoverdine I production decreased by 20% (Figures 1 and 2). However, the overexpression of *piuA* and *pvdS* into *P. aeruginosa* SR100216, by introducing plasmids ppiuA and ppvdS, did not cause a change in susceptibility (Table 2), and neither did by the addition of pyoverdine I (data not shown), suggesting a different resistance mechanism in SR100216 compared with SR100213. SR100216 showed an increased expression of *fecI* (Figure 1), which encodes a sigma factor that controls the expression of *fecABCDE* operon. As expected, expression of *fecA*, encoding an iron-citrate transporter, was also increased in mutant SR100216 (Figure 1). Overexpression of *fecI* and *fecA,* by introducing plasmids pfecI and pfecA into *P. aeruginosa* PAO1, confirmed that overexpression of these genes caused reduced susceptibility to cefiderocol (Table 2).

It is unclear why the overexpression of the iron-citrate transporter FecA would not result in an increased intracellular iron content, leading to significant reduced expression of *piuA* as observed for the *pvdS* mutant, but it could be due to insufficient amounts of ferric citrate in the medium. This hypothesis was supported by the fact that only addition of 500 μM, but not 50 μM, of citrate caused 4-fold and 2-fold increases in cefiderocol MIC against PAO1 and SR100216, respectively. A possible mechanism of why the increased expression of *fecI* caused the decreased susceptibility to cefiderocol, could be the saturation of TonB receptors, which are involved in both ferric citrate and ferric cefiderocol uptake, by excess amount of FecA. This possible mechanism is speculative and more detailed studies are needed.^26,32^

In conclusion, the mechanism of reduced susceptibility to cefiderocol in a *pvdS* mutant that resulted in the overproduction of pyoverdine was shown to be caused by a decreased expression of iron transporters including PiuA, which is involved in uptake of cefiderocol, rather than direct competition between cefiderocol and pyoverdine to chelate iron. On the other hand, for a *fecI* mutant that resulted in the overproduction of the iron-citrate transporter FecA, decreased expression of iron transporters, including those involved in uptake of cefiderocol, was not the mechanism of reduced susceptibility to cefiderocol. These data highlight different mechanisms by which bacteria adapt in trying to evade the action of cefiderocol by employing the complexities of iron haemostasis in bacteria. The importance of the investigation on the development of first-step resistance mechanisms has been reported,^33,34^ and continuous monitoring of resistance mechanisms is warranted.

A limitation of this study is that only *P. aeruginosa* PAO1 was used in this study, and it is unclear whether the observed mutations would cause decreased susceptibility of cefiderocol in other isolates. However, the same mutation on the upstream region of *pvdS* was observed in resistance acquisition studies using another clinical isolate in one of our studies (data not shown). Because we studied *P. aeruginosa* PAO1, we focused on *piuA* expression levels as this gene encodes the iron uptake transported involved in cefiderocol uptake in this strain,^5^ but a more comprehensive study of the regulation of all iron uptake systems may have been warranted to explain our findings, in particular for the *fecI* mutant. Another limitation of this study is that only the effect of pyoverdine was studied at 1 to 10 μM. There is a possibility that larger amounts of other iron-binding components might affect the iron metabolism in the bacteria as well, and iron homeostasis is complicated in the host as various iron-binding proteins or compounds exist at the infection sites. To address this, it will be necessary to better model the infection site more accurately to understand and quantify the risk to cause resistance in a clinical setting.

## Supplementary Material

dkag004_Supplementary_Data

## Supplementary data

Tables S1 and S2 is available as Supplementary data at *JAC* Online.
