# Supplementary material for: Understanding mechanisms of reduced cefiderocol susceptibility in pvdS and fecI mutants of Pseudomonas aeruginosa
Source: J Antimicrob Chemother. 2026 Jan 30;81(2):dkag004. doi: 10.1093/jac/dkag004 (PMC12856659; doi:10.1093/jac/dkag004)
Supplement: dkag004_Supplementary_Data [file dkag004_supplementary_data.docx]

# Supplemental Table S1. Primers for construction of recombinant plasmids and real time RT-qPCR for quantification of mRNA expressions

| Primers | Sequence |
| --- | --- |
| Cloning |  |
| Gm-In-F primer | CCCAGTTGACATAAGCCTGTTCGG |
| Gm-In-R primer | GTAAATTGTCACAACGCCGCGGCCGC |
| pvdS-HindIII-F | AATTAAGCTTATGTCGGAACAACTGTCTACCC |
| pvdS-BamHI-R | CCGGGGATCCTCAGCGGCGGGCG |
| fecI-HindIII-F | AATTAAGCTTGTGTCGAGCGCCGACCTC |
| fecI-BamHI -R | CCGGGGATCCTCATGGCAGCTCGGCGAAG |
| fecA-HindIII-F | AATTAAGCTTATGTCCCCGTCACGCGC |
| fecA-BamHI -R | CCGGGGATCCATGTCCCCGTCACGCGC |
|  |  |
| Real time RT-qPCR |  |
| uvrD-F | CTACGGTAGCGAGACCTACAACAA |
| uvrD-R | GCGGCTGACGGTATTGGA |
| piuA-f: | TCTCAGCACCGATGATATGC |
| piuA-r: | TTGTCGTTCAGGTCATGCTC |
| pvdS-F | GCAGATCACTTCGTCGTTCA |
| pvdS-R | AGTTGATGTGCGAGGTTTCC |
| FecA-F | TGGAACCTCTACGCCAACA |
| FecA-R | TCGAAGTTGATCAGGAAGGC |
| FecI-F | TTCGTCAAGGTCCTGGTTTC |
| FecI-R | TCTCTTCGCTGGGGACTT |
|  |  |
| Restriction sites in the primer sequences are underlined. | |

**Supplemental Table S2.** Determination of relative amounts of pyoverdine I produced by PAO1 and *pvdS* and *fecI* mutants

The peak areas of pyoverdine I (*m/z 1333.7*) and pyoverdine II (*m/z 1092.5*) were determined by MALDI TOF mass spectrometry in supernatants of strains PAO1, SR100213 (*pvdS* mutant) and SR100216 (*fecI* mutant) supplemented with 50 μM of pyoverdine II (internal standard). The relative amounts of pyoverdine I of SR100213 and SR100216 were compared to that of strain PAO1.

| Strain | Dilution | Peak area of pyoverdine I | Peak area of pyoverdine II | Relative amount of pyoverdine I^1^ | Cell density (OD_600_) | Average ± SD of relative amount of pyoverdine I^2^ |
| --- | --- | --- | --- | --- | --- | --- |
| PAO1 | 1 | 1561.5 | 9531.9 | 0.16 | 0.47 | 1.00 ± 0.16 |
|  | 1 | 1662.0 | 7311.1 | 0.23 |  |  |
|  | 1 | 821.4 | 4003.1 | 0.21 |  |  |
| SR100213 | 5 | 3491.0 | 13558.8 | 1.29 | 0.51 | 7.15 ± 1.82 |
|  | 5 | 4404.9 | 16253.0 | 1.36 |  |  |
|  | 5 | 2203.3 | 5511.3 | 2.00 |  |  |
| SR100216 | 1 | 528.9 | 4691.2 | 0.11 | 0.52 | 0.82 ± 0.26 |
|  | 1 | 1045.0 | 4966.1 | 0.21 |  |  |
|  | 1 | 1602.0 | 7502.3 | 0.21 |  |  |

1: Determined by Peak area of pyoverdine I × dilution/Peak area of pyoverdine II

2: Relative amount of pyoverdine I/OD_600_ compared to strain PAO1
